# Medical Opinion and Movement

**Published:** 1910-11-26

**Authors:** 


### Medical Opinion and Movement.

### Parathyroid Glands and Sudden Death.

Many recent researches have tended to show the importance of the parathyroid glands to life, and according to some cases observed by Drs. Grosser and Belke lesions of these glands would appear to be responsible for the occurrence of sudden death in infants. Three cases are reported by them. The first, an infant aged three months and weakly, died suddenly in the course of a few minutes. Two other children of the same family had died of convulsions at the ages of seven weeks and two and a half months respectively. The second, aged three months, was suffering from broncho-pneumonia and suddenly developed cyanosis and dyspnœa, and died in three-quarters of an hour. The third, also aged three months and very thin, had suffered for three weeks from muscular spasms and died suddenly in convulsions. At the autopsies only one organic lesion of any importance was discovered, and that was common to all three cases —intense hæmorrhages were found in the parathyroid glands, destroying the greater part of the glandular tissues, and compressing the rest so as to form inter-communicating hæmorrhagic pseudocysts. These hæmorrhages were for the most part old, and the glandular tissue still present showed proliferation of the interstitial tissue, which was also found in the thyroid of the first case. In the first two cases three of the parathyroid glands were affected, and in the third all four glands were involved.

### Trauma and Muscular Atrophy.

It is already well known that the muscles of an injured limb atrophy, and that those of the corresponding healthy limb, probably in consequence of disuse, also undergo a certain amount of wasting; but according to the observations of Dr. P. Piet on ninety cases of fracture or other injury to the limbs, the healthy limb underwent approximately the same amount of wasting as the corresponding injured limb in at least 60 per cent. of the cases. In view of this fact, in order to estimate the amount of wasting in an injured limb it is quite fallacious to take comparative measurements with the healthy limb. In these cases both limbs were measured at the time of the accident, and subsequent measurements were made periodically at the same time of the day and, as far as possible, under the same conditions. Daily variations may amount to as much as 4 centimetres. This is, however, an extreme figure, but under ordinary circumstances the daily variation is about 2½ centimetres. The amount of wasting of the injured limb attains a maximum at the end of three days, the wasting of the healthy limb reaches a maximum generally at a later date, but they both commence to recover to the normal at the same time. The increase in volume is especially marked when the limb first begins to functionate normally without any support, and often attains a volume in excess of its previous condition, or of its subsequent condition, when all sign of trauma has passed off. The author attributes this symmetrical wasting to a reflex action of the medullary centres upon the circulatory system of the two limbs.

### The Only Child.

At a meeting of the Medical Society of Vienna Dr. J. Friedjung read an interesting paper on what he calls the pathology of the "only child." In this communication he draws attention to special morbid symptoms and conditions which certain children develop, and in particular the much favoured and petted children, of whom the only son is a typical example. These children are constantly ailing, they develop obstinate morbid symptoms difficult to explain, and the ordinary children's diseases take with them an atypical course. The author has collected one hundred cases of such children, forty-five boys and fifty-five girls, aged two to ten years. Of these hundred, eighteen were seriously neuropathic, sixty-nine were moderately so, and only thirteen were healthy, whereas of one hundred children from families of several offspring, sixty-nine were healthy and thirty-one neuropathic. The neuroses of these children presented the characteristics of neurasthenia and of hysteria. Besides a typical development in the character, seventy-five showed a condition of mental anxiety more or less pronounced, forty-nine had sleep disturbances, eight had fits of nocturnal fright, thirty-two suffered from malnutrition, anorexia, often associated with vomiting, twenty were afflicted with habitual constipation, and in many cases this alternated with catarrh of the lower bowel; forty-eight had lichen urticatus, and many suffered from enuresis. The author is of the opinion that the cause of all these troubles lies in the excess of care and tenderness with which the "only child" is surrounded.

### Pericardial Paracentesis.

Numerous observations have established the fact that puncture of the pericardium to the left of the sternum is liable to wound the heart when the trocar is inserted immediately external to the sternum, or to injure the pleura if the pericardium is approached at its left externo-inferior angle. The pleura is also exposed to injury if the trocar is inserted to the right of the sternum. In view of these difficulties Dr. A. Gras has studied the conditions of a pericardial effusion and the topographical modifications which result in regard to the heart and the adjacent organs. He injected the pericardium of fifteen cadavers with gelatine to the extent of about 600 grammes, placing the bodies in different positions during the cooling of the fluid, and then performed paracentesis by different methods. He found that the injected solution accumulates chiefly in the posterio-inferior part of the pericardium, where it attains a depth of 2 to 4 centimetres. At the right and left margins also it amounts to about 2 centimetres, but the anterior surface of the heart is only covered by a thin layer, leaving more or less of the cardiac muscle and the interventricular sulcus in direct contact with the parietal pericardium. Thus the heart is raised upwards and forwards, and floats on the effusion. In regard to the pleuræ, the left leaves the sternum at the fifth intercostal space, and is 3 centimetres distant at the seventh. The right pleura is at the sternal margin in the fifth space, and leaves it slightly at the sixth, but these relations show considerable variations. As puncture of the heart is more serious than injury to the pleura, the author considers that the site of election for pericardial puncture is the sixth space to the right of the sternum. The trocar should be inserted right against the sternum, the point being directed upwards and inwards. In cases in which the effusion is very small, the right fifth intercostal space is to be preferred, the trocar being carried in against the sternum as before and in the direction of the left nipple. These cases, however, rarely call for any such intervention.

### Intubation of the Colon.

Dr. H. W. Yates has read a paper before the American Association of Obstetricians and Gynæcologists which embodies much patient work. Starting with the belief that to pass a tube per rectum into the colon is not at all impossible, as certain observers have maintained, he conducted experiments with many different kinds of tube and checked them by *x-ray* observations and injections of bismuth emulsion. He was speedily forced to abandon his preconceived opinions, and to adopt the conclusions summarised thus: Seldom, if ever, are soft rubber tubes admitted into the normal colon. When an endeavour is made to force the tube upward, even by the gentlest manipulations, it is found to coil itself up in the rectum, and there do positive harm because of pressure, irritation, and consequent inability to retain the enema. In perhaps half the cases it is impossible to tell when the tube is coiling itself even when we suspect it. Colon tubes as such are of no value, because they do not reach the colon, and they are mischievous in proportion as we endeavour to force them higher. Water or fluid injected four or five inches into the rectum is carried upwards into the colon, and may be found at the cæcum in ten minutes. There is good reason to believe that a reversed peristalsis is set up when fluids are injected into the rectum. The introduction of a tube of more than 5 inches for colonic irrigation or therapeutic enemata is useless and likely to defeat the object desired.

### Vincent's Angina.

Two interesting and novel cases of a lesion, which is possibly not so rare as unsuspected, are reported by Dr. H. Arrowsmith in the *Journal of Laryngology, Rhinology, and Otology*. The condition described is a primary laryngitis due to the bacillus of Vincent's angina, without involvement of the fauces, analogous with primary laryngeal diphtheria. This is a disease never hitherto recorded in medical literature. In one case a man of twenty-six came under notice complaining of discomfort in the throat, with increasing hoarseness and dyspnœa, of one week's duration. Pulse, temperature, and chest were normal; there was slight swelling of the neck externally. The epiglottis and arytenoids were œdematous. Twenty-four hours later the dyspnœa became so acute that tracheotomy was necessary. The tube was removed in four days, and three weeks later the patient was apparently quite cured; but after another three weeks he returned with a recrudescence of the same symptoms, and with pus discharging from the site of the tracheotomy, which had spontaneously broken down. The pus contained immense numbers of the fusiform bacilli and spirilla of Vincent. Once more tracheotomy had to be done; the tracheal contents and the sputum contained the specific germs in almost pure culture. Finally thyrotomy was performed, from which the patient recovered (after a uræmic attack), with a good deal of stenosis. Syphilis was excluded, both by the negative results of treatment and by the Wassermann test.

### The Mongolian Blue Patch.

One of the classical signs of mongolism is the presence of a blue patch in the skin on the buttock or somewhere in the sacral region. It is present at birth, and gradually fades, disappearing at about six or seven years if the child survives to that age. It has been found in about 80 to 90 per cent. of the infants of the Mongolian races, and it also occurs (less frequently) among negro tribes. Among pure Europeans it is found in about 0.5 per cent. of children, and its presence does not necessarily indicate any tendency to Mongolian idiocy, though it is commonly seen in that disease. MM. Planchu and Rendu record a case recently in the *Lyon Médicale*, and H. Carnot records two more in a *Thèse de Paris*, which deals with the whole subject of these curious blue patches. Histologically the patch is due to the presence of numerous richly pigmented cells situated in the deep layers of the dermis. The cells are grouped round the capillaries, and are thought to be due to proliferation of the endothelium. The occurrence of these patches in European children is ascribed not to mixed blood but to atavism, or a throw-back to an original ancestor of both races, probably of dark or black skin. It is said that similar patches occur in some species of monkeys.

### Sleeping-Sickness in Whites.

The last number of the *Bulletin of the Sleeping-Sickness Bureau* contains an article on sleeping-sickness in whites, from which we gather that complete records have now been obtained of fifty cases of the disease occurring in Europeans. In addition to enlargement of the cervical glands, which seems a fairly constant symptom, fever was recorded in forty cases, erythema of the skin in thirty-one, loss of strength in twenty-nine, tachycardia in twenty-five, somnolence in twenty-three, œdema in twenty-two, enlarged spleen in nineteen, anæmia and headache each in eighteen, epileptiform fits in seventeen, tremor in fifteen, character changes in twelve, itching in eleven, hyperæsthesia and urinary or fæcal incontinence each in ten, enlargement of the liver and eye symptoms each in nine, delusions in seven, insomnia in six, vomiting in five, melancholia, epistaxis, and pains in the feet each in four, paresis of lower limbs and dermatographia each in three, painful swelling on the ankle, sexual impotence, and hemiplegia each in two cases. Giddiness, diarrhœa, loss of hair, phlebitis, facial paralysis, localised œdema, and orchitis are each noted once. Auto-agglutination was recorded in six instances. Of the fifty patients thirty are known to be dead, eleven survive, and the fate of nine is uncertain. The treatment used was varied, but in no cases was it intensive as it would be nowadays. The recovery of a number of them must be attributed at least as much to the natural resistance of the body as to the treatment employed. It would seem that there is good hope of ultimate recovery in cases which have survived more than four years after the diagnosis has been established.

### Ophthalmia Nodosa.

An affection of the eye due to the entrance into the eyeball of the hairs of certain caterpillars is known under the name of ophthalmia nodosa. It is most commonly met with among those who work in the fields and woods, but people vary in susceptibility, and it is not always possible to obtain a history of direct contact. Several species of caterpillar are implicated, but the chief offender would seem to be the Bombyx rubi. W. H. Parker, of Detroit, in the *Journal of the American Medical Association*, describes the condition. After reviewing the literature on the subject, he states that the irritative symptoms follow closely upon the accident. The sensation is that of a foreign body in the eye, accompanied by lachrymation, itching, œdema of the lids and conjunctiva and pericorneal injection. This condition continues for four to eight days, after which the hairs penetrate the tissues, the first tubercular nodules appear, and the disease becomes typical. The condition is not primarily of bacterial origin, for it has been proved that formic acid is present in the glandular secretion of the hair. After a time it tends to subside, though relapses are liable to occur. The disease may be prevented if the hairs are removed before they can penetrate into the tissues or are rubbed in. It is at times necessary to resort to curetting to relieve the condition. The author reports a case which lasted several months, and was only cured by operation, which brought to light the embedded hair. His article contains a list of the cases of the disease reported in the literature.

### Heroin-Chloroform Anæsthesia.

Dr. P. Volta has been experimenting to determine the value of heroin as an adjunct to anæsthesia induced by chloroform, and publishes his results in the *Gazetta degli ospedale e delle Clineche.* Half an hour before being taken to the theatre 1 c.c. of a sterilised 5 per cent. solution of heroin hydrochloride was administered subcutaneously. In this way a profound regular and persistent narcosis was obtained subsequently with chloroform. Patients were always quiet fifteen minutes after the heroin injection, without showing marked symptoms of somnolence. Respiration was deep and regular, and there were no marked alterations in the character of the pulse. The author thinks that the administration of heroin is not contra-indicated even in heart disease, since the heart and the respiratory apparatus have not been adversely influenced in the cases he has treated in this way. The deep respirations induced by the drug ensure a rapid and regular intake of chloroform vapour into the lungs, and many of the usual complications of the induction of anæsthesia are avoided, such as asphyxia from excessive mucus secretion, and syncope from reflex causes. Anæsthesia approaches in type normal sleep, respiration is slow but deep and regular, the pulse is full, and the temperature slightly above the normal. This practically ideal state persists without alteration to the end of the induction. Vomiting is rare, and when present is slight. The patient rapidly recovers from the anæsthetic, and in about an hour has completely overcome the condition of drowsiness which is certainly not favourable to the regaining of strength.

### The Wassermann Reaction in Leprosy.

Montesanto and Sotiriadès publish in *La Presse Médicale* the results of performing Wassermann's reaction in a series of forty-eight cases of leprosy. A positive result was obtained most frequently in the tuberculous variety of the disease, their figures amounting to 6.29 per cent., whereas in mixed forms the figures were 5 per cent., and in the anæsthetic type of the disease 1.66 per cent. They are inclined to think that the reaction tends to show a positive result more frequently in the advanced stages of the disease, where the lesions are well marked, than in its early stages, and if their supposition is correct it will account for the differences met with in their series. Analogous results to these were recently reported by Meier to the Second Leprosy Conference.

### The Purification of Oysters.

At a recent meeting of the Académie des Sciences de Paris, M. Henneguy reported some experiments made at the Concarneau laboratory by M. Fabre-Domergue with reference to the methods that should be used to prevent the accidents caused by contaminated oysters. It is a matter of common knowledge that those matured in beds of which the water is polluted by sewage are apt to be the means of disseminating typhoid fever. The author has been able to convince himself that it is possible to render these shell-fish harmless, even after they have been reared in the most insanitary of surroundings, by placing them for a fortnight in filtered water before selling them to the public. He states, moreover, that this form of "quarantine" in pure water has no evil effects on the quality of the oyster, and that it remains as fat and as succulent as before its removal from its insanitary surroundings.

